# Expression of Transcription Factor *CREM* in Human Tissues

**DOI:** 10.1369/00221554211032008

**Published:** 2021-07-14

**Authors:** Heidi Kaprio, Vanina D. Heuser, Katri Orte, Mikko Tukiainen, Ilmo Leivo, Maria Gardberg

**Affiliations:** Department of Pathology, Institute of Biomedicine, Turku University Hospital and University of Turku, Turku, Finland; Department of Pathology, Institute of Biomedicine, Turku University Hospital and University of Turku, Turku, Finland; Department of Pathology, Institute of Biomedicine, Turku University Hospital and University of Turku, Turku, Finland; Auria Biobank, Turku University Hospital and University of Turku, Turku, Finland; Department of Pathology, Institute of Biomedicine, Turku University Hospital and University of Turku, Turku, Finland; Department of Pathology, Institute of Biomedicine, Turku University Hospital and University of Turku, Turku, Finland

**Keywords:** cancer, CREB, CREM, cyclic AMP element modulator, fusion gene, ICER, immunohistochemistry, normal tissue, transcription factor

## Abstract

Cyclic AMP element modulator (CREM) is a transcription factor best known for its intricate involvement in spermatogenesis. The *CREM* gene encodes for multiple protein isoforms, which can enhance or repress transcription of target genes. Recent studies have identified fusion genes, with *CREM* as a partner gene in many neoplastic diseases. *EWSR1-CREM* fusion genes have been found in several mesenchymal tumors and in salivary gland carcinoma. These genes encode fusion proteins that include the C-terminal DNA-binding domain of CREM. We used a transcriptomic approach and immunohistochemistry to study the expression of CREM isoforms that include DNA-binding domains across human tissues. We found that CREM protein is widely expressed in almost all normal human tissues. A transcriptomic analysis of normal tissues and cancer showed that transcription of *CREM* can be altered in tumors, suggesting that also wild-type CREM may be involved in cancer biology. The wide expression of CREM protein in normal human tissues and cancer may limit the utility of immunohistochemistry for identification of tumors with *CREM* fusions:

## Introduction

Cyclic AMP element modulator (CREM) is a transcriptional regulator protein that belongs to the cAMP response element–binding protein (CREB) transcription factor protein family. This is a family of basic domain-leucine zipper proteins with a capacity to bind to the cAMP response element (CRE), which is a sequence present in the regulatory region of a large number of target genes.^1,2^ The *CREM* gene has several promoters that give rise to multiple isoforms. Different isoforms of CREM can promote or repress expression from the CRE. The gene product from its promoter P2 represses transcription from the CRE and is denoted as inducible cAMP early repressor (ICER).^3^ CREM protein is known to be highly expressed in the testis, where it is essential for spermatid development.^2,4^ A few studies have found the functional roles of CREM in the central nervous system (CNS). CREM expression has been found to increase in the rat CNS after neuronal damage, proposing a role in CNS injury and repair.^5^ It has also been discovered to have a role in mediating impulsive behavior and addiction, as well as in regulating the spinal morphology and neuroplasticity.^6^

A few studies have linked altered CREM protein expression to cancer, more specifically to prostate and esophageal carcinoma.^7,8^ Recently, the *CREM* gene has been found as a fusion gene partner in several types of tumors. The most commonly found *CREM* rearrangement is the EWS RNA binding protein 1 (*EWSR1*)-*CREM* fusion gene. It is recurrent in several types of neoplasia such as mesenchymal tumors, in hyalinizing clear cell carcinoma of salivary glands, and, most recently, in a malignant epithelioid neoplasm with predilection for mesothelial-lined cavities.^9–13^ The *EWSR1-CREM* fusion genes encode fusion proteins with an N-terminal *EWSR1* transactivation domain and a C-terminal DNA-binding domain of CREM.^14^

Detection of the fusion protein or neoexpression of the CREM protein could potentially serve as a surrogate for genetic testing, if the expression of wild-type CREM protein in tissues would be low. Furthermore, a basic description of *CREM* expression in tissues and cell types would be instrumental for studying the functional roles of CREM in both physiological and disease-associated processes. Despite this, the expression of CREM protein has not been studied using an antibody that targets the C-terminal DNA-binding domains of CREM, which are present in virtually all CREM isoforms. To bridge this gap in knowledge, we herein characterize the expression of *CREM* in human tissues, using publicly available transcriptome databases, and on protein level using immunohistochemistry (IHC) of tissue microarrays (TMAs) built for this purpose using an antibody targeting the C-terminal part of most CREM isoforms. With the expression pattern of CREM portrayed, we also test whether CREM IHC could be used as an indicator of the presence of *EWSR1-CREM* fusion gene in low-grade mucoepidermoid carcinoma (MEC).

## Materials and Methods

### Cell Culture

The commercially available human skin melanoma CHL-1, the human embryonic kidney HEK-293, and the human prostate carcinoma PC-3 were obtained from American Type Culture Collection (Manassas, VA). CHL-1 and HEK-293 cells were maintained in DMEM supplemented with 10% fetal bovine serum (FBS), 5 mM ultraglutamine, and 100 U/ml penicillin-streptomycin (Gibco; Carlsbad, CA), whereas PC-3 cells were cultured in RPMI medium with the same supplements.

### CREM Knockdown

The CREM transcripts were knocked down in CHL-1, HEK-293, and PC-3 cells using 50 nM of human CREM small interfering RNA (siRNA) (sc-37700; Santa Cruz Biotechnology, Santa Cruz, CA) containing a pool of three to five target-specific 19–25 nucleotide sequences in length. A mix of three different non-targeting siRNAs (sc-37007, sc-44230, and sc-44231; Santa Cruz Biotechnology) was used as control. Cells were transfected using Dharmafect 4 transfection reagent (Dharmacon Research) according to the manufacturer’s instructions. The knockdown efficacy was examined 48 hr after transfection by Western blotting. The experiments were repeated at least 3×.

### Western Blotting

Cells treated with CREM siRNA or control siRNA were lysed in a radioimmunoprecipitation assay buffer supplemented with protease inhibitors. Samples were normalized for protein concentration, and equal amounts of material in the Laemmli sample buffer were subjected to SDS-PAGE and transferred to nitrocellulose filters. For immunoblotting, the mouse monoclonal anti-CREM antibody (clone 3B; Novusbio, Littleton, CO) and the rabbit monoclonal anti-EWSR1 antibody (ab133288; Abcam, Cambridge, UK) were incubated overnight in a cold room at 1∶1000 dilution in 5% BSA/TBS/Tween 0.1%. After that, the membrane was incubated for 1 hr at room temperature with a mix of goat anti-rabbit StarBright Blue 520 and goat anti-mouse StarBright Blue 700, and hFAB rhodamine anti-GAPDH (Bio-Rad, Hercules, CA, USA) antibodies at 1:1000 dilution. Equal protein loading was evaluated by the housekeeping protein glyceraldehyde-3-phosphate dehydrogenase (GAPDH). The membrane was washed 3× with TBST between incubations. Bound proteins were detected by fluorescence using ChemiDoc Gel Imaging System (Bio-Rad).

### Human Tissue Samples

To explore CREM protein expression in normal human tissues, we built a TMA of 39 different human tissues in collaboration with Auria Biobank. For this, formalin-fixed paraffin-embedded (FFPE) archival tissue samples from Auria Biobank were used. All procedures were followed in accordance with the principles of the Helsinki Declaration of 1975. The study was authorized by the Hospital District of Southwest Finland (decision T3/2019) and the Auria Biobank steering committee (decision AB19-2770).

Four patient samples of each location/tissue type were included, apart from male and female reproductive tissues, in which two samples each were included. The tissue samples had originally been taken surgically for diagnostic histological analysis at the Turku University Hospital Department of Pathology, except for the cerebrum, cerebellum, and heart samples, which were taken at autopsy. To identify potential samples, Auria Biobank sample and data archives were probed with SQL searches using SNOMED codes. Criteria for the samples were patients aged 16–75 years at the time of sampling and a desired topography. Reproductive tissues were from patients of the following age (years): fallopian tube fimbriae, 41–49; myometrium, 35–42; ovary, 30–34; secretory phase endometrium, 35–41; proliferative phase endometrium, 40–42; breast, 37–41; testis 49–62; and prostate, 70–73. H.K. and M.G. selected cases with essentially normal histology and made 1.5 mm diameter annotations to hematoxylin and eosin–stained scanned slides using CaseViewer software (3DHistech; Budapest, Hungary). Corresponding FFPE tissue cores were transferred into TMA blocks using TMA Grand Master (3DHistech). Bone marrow and breast tissue were used as whole sections due to technical reasons (adipose tissue and bone section poorly from a TMA block). Skin samples were used as whole sections to find a sufficient number of melanocytes for analysis. Bone marrow samples had been decalcified before paraffin embedding.

### Transcriptomic Analyses

Publicly available databases were used to examine the expression of CREM mRNA. Normal tissue expression was investigated using the GTEx Portal V8 Release (GTEX database). For comparison of expression in normal tissue and cancer, we used the Gene Expression database of Normal and Tumor tissues 2 (GENT2; available at http://gent2.appex.kr/gent2/). Student’s *t*-test analysis was conducted using SPSS version 26 (IBM Corp.; Armonk, NY).

### Immunohistochemistry

Paraffin blocks were sectioned at 4 µm. Immunostaining was performed using a LabVision autostainer (Thermo-Fisher Scientific, Waltham, MA). For antigen retrieval, Tris-EDTA pH 9.0 and microwave heating were used. Endogenous enzymes were blocked with hydrogen peroxidase, and a pre-protein block was done using Draco antibody diluent (WellMed AD125). The TMA sections were incubated for 60 min at room temperature with the mouse monoclonal anti-CREM antibody (clone 3B; Novusbio; 1:000 dilution) in Draco antibody diluent. The sections were then washed with 0.05 M Tris-HCl and treated with Orion II step detection system goat anti-mouse/rabbit secondary antibody [horseradish peroxidase (HRP), WellMed T100 HRP] at room temperature for 30 min. Counterstaining was done with Mayer’s hematoxylin, and finally, the sections were mounted with PERTEX.

### Evaluation of IHC

The CREM-stained TMA slides were scanned using a Panoramic 250 Flash scanner (3DHistech) and histologically evaluated CaseViewer software (3DHistech). H.K. took pictures of each tissue sample from a representative area containing at least 100 cells of desired type (or if needed, more pictures were taken and the results were summed) and used ImmunoRatio software to calculate positive nuclear staining.^15^ Staining intensity was evaluated by H.K. and M.G. independently, and discrepancies were discussed and evaluated together. Intensity was scored using a four-tier grading system: 0 (negative), 1 (mild), 2 (moderate), and 3 (strong). The dominant staining intensity was evaluated and the percentage of positive nuclei was calculated for each sample separately.

## Results

### Validation of Antibody

**Figure 1.**
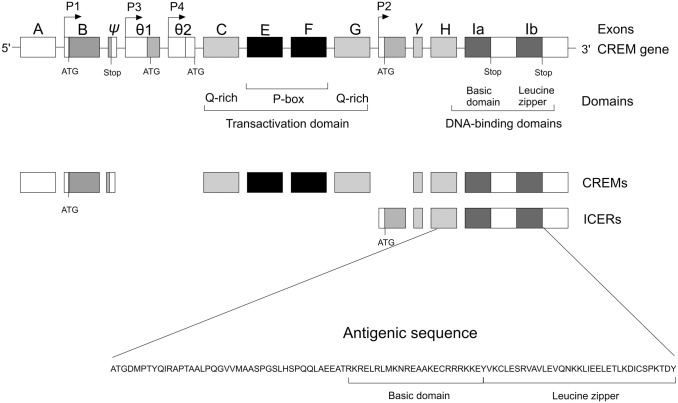
Schematic presentation of *CREM* gene, CREM/ICER proteins, and antibody target sequence. Exon structure of the *CREM* gene showing *CREM* alternatively used promoters P1, P3, P4, and ICER promoter P2. Arrows indicate the start of transcription. ATG indicates the start of translation and STOP codons. Exon size and intron distance are not to scale. The corresponding functional domains of CREM and ICER protein are noted below. The transactivation domains consist of exons E and F encoding the regulated phosphorylation [P-box; also called kinase-inducible domains (KID)] and the two glutamine-rich (Q-rich) exons involved in basal transactivational activities, which are absent in ICERs. The DNA-binding domains are exons H and I that encode the basic regions required for DNA recognition and the leucine zipper dimerization domains, respectively. Exon I can be alternatively spliced in Ia and Ib. Antigenic sequence targets amino acids 201–300 of protein sequence, corresponding to exons H and I, that includes DNA-binding domain.^16^ Abbreviations: CREM, cyclic AMP element modulator; ICER, inducible cAMP early repressor.

First, we wanted to ensure that the antibody we used specifically detects the CREM protein. This commercially available mouse monoclonal anti-human CREM antibody (clone 3B; Novusbio) had been generated using a target sequence containing amino acids 201–300 of the protein, which includes the DNA-binding domain of the CREM protein. A schematic presentation of the *CREM* gene, protein, and the antibody target sequence is presented in Fig. 1. This antibody could detect 28 of the 29 CREM isoforms with sequence identity of 85–100%. The sequence homology with members of the same transcription family CREB and ATF is low (67% and 57%, respectively).

**Figure 2.**
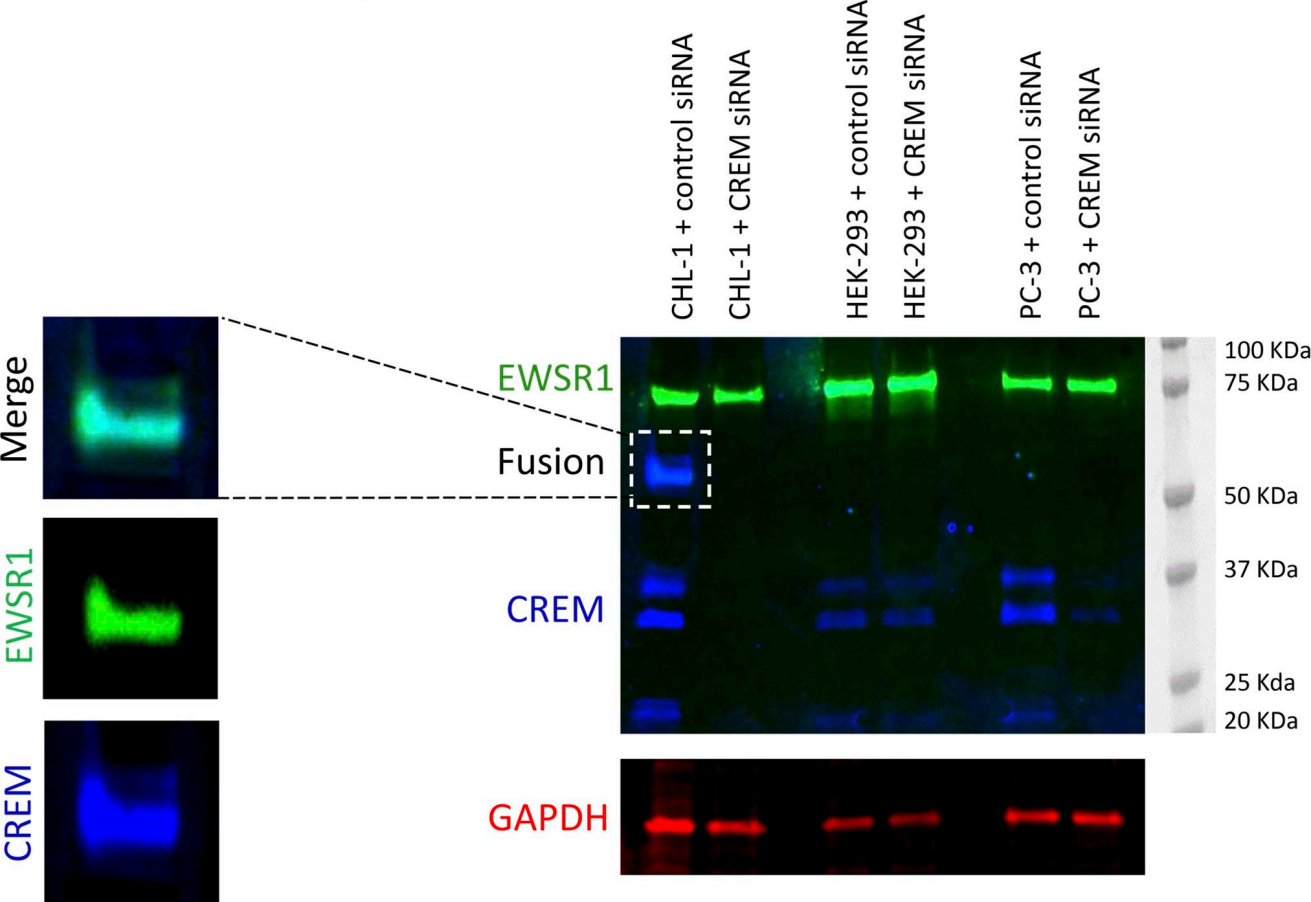
Validation of the antibody used for immunohistochemical stainings using Western Blotting and siRNA-mediated knockdown of CREM expression. Western blotting using cell lines CHL-1, HEK-239, and PC-3 shows bands of expected size using the CREM antibody (between 20 and 37 KDa depending on isoform). After transfection using CREM-targeting siRNA, the bands are markedly weaker, indicating that the knockdown of CREM is successful and that the antibody specifically detects CREM (blue bands). The bands are of different size than those seen using CREB (expected size of 43 KDa; data not shown). After CREM siRNA treatment, only bands detected by anti-CREM are weaker, confirming that this antibody detects CREM specifically. EWSR1-CREM fusion (≈55 KDa) in the CHL-1 cell line was detected with both anti-CREM and EWSR1 antibodies (superpositions of blue and green). Endogenous EWSR1 (green bands) was not affected by CREM knockdown, and GAPDH shows equal amounts of protein loading. Molecular marker: Dual Color Precision Plus Protein standards (Bio-Rad). Abbreviations: CREM, cyclic AMP element modulator; GADPH, glyceraldehyde-3-phosphate dehydrogenase; siRNA, small interfering RNA.

First, we performed Western blotting using CREM antibody on cell lysates from human skin melanoma CHL-1, human embryonic kidney HEK-293, and prostate cancer PC-3 cell lines. The cells had been treated with CREM-targeting siRNA or control siRNA. Two or three major bands of expected size (≈37, 30, and 20 kDa) were detected in the immunoblots of all control cell line lysates (Fig. 2). Furthermore, we wanted to test whether the CREM antibody would detect an EWSR1-CREM fusion in the CHL-1 cell line, which is known to harbor this rearrangement.^17^ Western blotting using the anti-CREM or anti-EWSR1 antibody detected a band (≈55 kDa) not corresponding to the predicted size of CREM. The two secondary antibodies were then labeled with different fluorescence labels, and in double immunofluorescent blotting they co-localized in the same band identifying it as the EWSR1-CREM fusion protein. The fusion protein band was markedly weaker after CREM siRNA treatment, indicating successful knockdown. Also the bands corresponding to wild-type CREM protein were weaker in all three cell lines after CREM knockdown. Endogenous EWSR1 (which is ubiquitously expressed) was not affected by CREM knockdown. Taken together, these findings agree that the CREM antibody specifically detects CREM protein.

### Analysis of CREM mRNA Expression in Human Tissue From Databases

To get an overview of *CREM* mRNA expression that we could later compare protein expression with, we studied the expression of *CREM* mRNA in different human tissues using the GTEx database. The expression was calculated from a gene model with isoforms collapsed to a single gene (the GTEX database can also be used to investigate isoform-specific expression).

**Figure 3.**
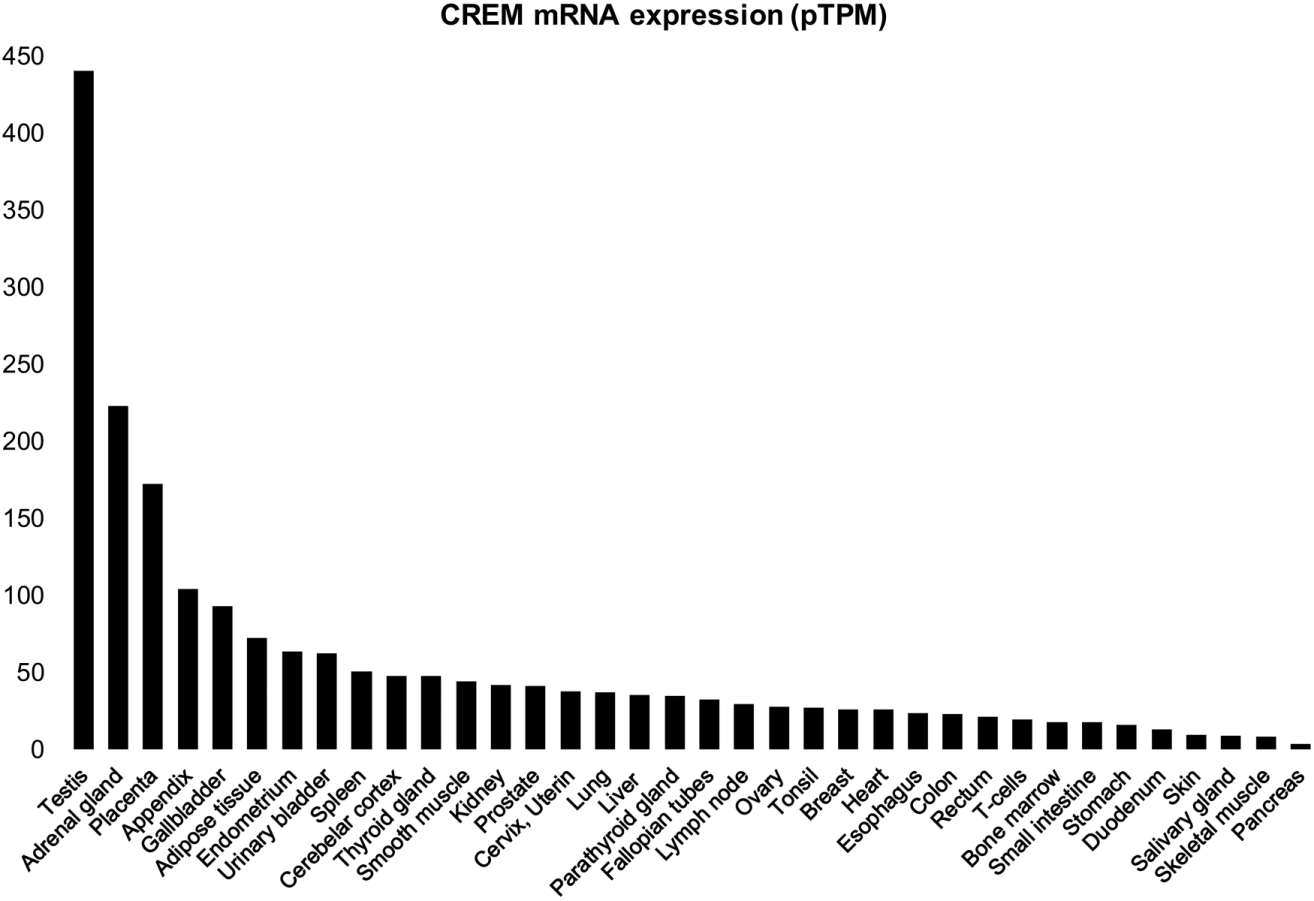
Analysis of CREM mRNA expression in different tissues using the GTEX database. Expression of CREM mRNA in different human tissues, arranged from the highest expression to the lowest expression. Values are shown in TPM (transcripts per million). No other normalization steps have been applied. Abbreviations: CREM, cyclic AMP element modulator.

In this analysis, the highest expression of CREM was seen in the testis, followed by the adrenal gland, the placenta, and the appendix. Most tissues expressed CREM at moderate or low levels. The least expression was seen in the pancreas, followed by the skeletal muscle, the salivary gland, and the skin (Fig. 3).

### Immunohistochemical Analysis of CREM in Human Tissues

**Figure 4.**
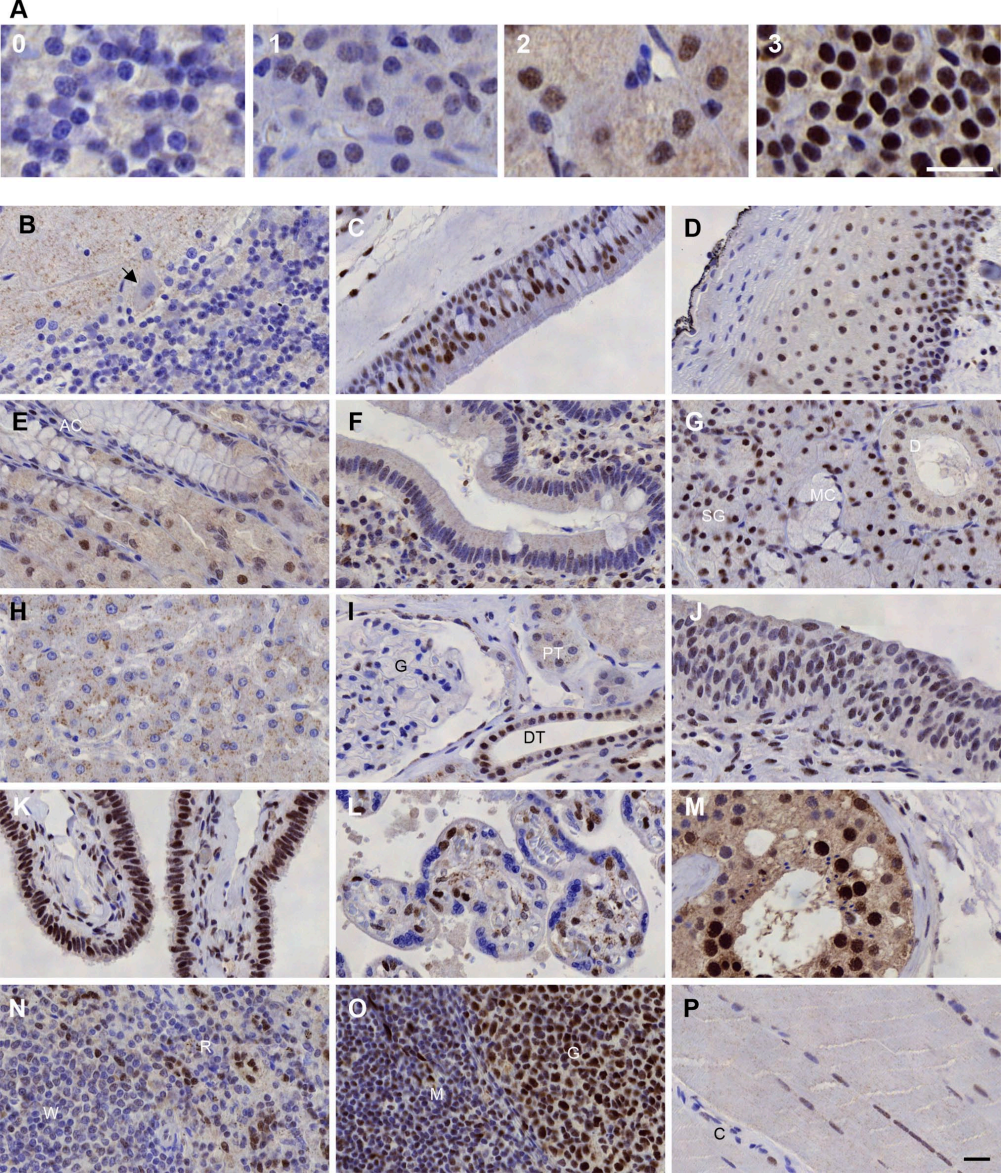
Immunohistochemical detection of CREM in human tissues. (A) The four-tier immunohistochemical evaluation scheme for CREM stainings: 0 negative (cerebellum), 1 weak (pancreas), 2 moderate (stomach), and 3 strong (parathyroid). Scale bar = 20 µm. (B) In the cerebellum, we detected virtually no nuclear staining. Black arrow indicates a Purkinje cell on the border of the molecular and granular cell layers. (C) In the respiratory epithelium of the nasal cavity, 60–69% of cells were positive with dominantly moderate staining intensity. The negative cells were usually of goblet cell type. (D) The palate squamous epithelium had a gradient expression profile. (E) The apical cells (AC) of the gastric corpus were negative, whereas the glandular cells expressed CREM. (F) The duodenal stromal cells had weak to moderate staining intensity, whereas enterocytes and goblet cells were predominantly negative near the crypts as seen here. Apical enterocytes had more CREM expression. (G) In the submandibular gland, the mucous glands (MG) were weaker and more narrow in their CREM expression of the ducts (D) and serous glands (SG). (H) The hepatocytes of the liver had negative nuclei and a moderate granular cytoplasmic staining. (I) In the kidney glomerulus (G), only just over half of the nuclei stained positive, and the proximal tubules (PT) and distal tubules (DT) had wider expression. (J) In the urinary bladder epithelium, most of the nuclei had CREM expression. (K) The ciliated columnar cells of the fimbriae epithelium were among the strongest staining cell types. (L) In the placenta, the tertiary villi had negative syncytiotrophoblasts lining the villi, whereas half of the cytotrophoblasts had moderate CREM expression. (M) In the testis, seminiferous tubules (T) had different nuclear staining intensities, from negative to strong. (N) The white pulp (W) in the spleen was mostly negative, whereas in the red pulp (R) we observed clear staining of the sinusoidal cell nuclei. (O) In the lymph node cortex, the germinal center (G) had a near-uniform positive CREM expression, whereas interestingly the mature cortical lymphocytes (M) remained almost negative. (P) Skeletal muscle nuclei had a wide weak to moderate staining pattern. Here, the capillaries and supporting tissue are nearly negative (C). The scale bar in picture O is 20 µm; all images are of the same magnification. Images of the 15 tissues in B–P in smaller magnification are available as supplements. Abbreviation: CREM, cyclic AMP element modulator.

To gain detailed knowledge of which specific cell types in various organ systems express the CREM protein, an immunohistochemical analysis was performed on TMAs with several samples of 39 normal human tissues. Staining intensity was divided into four groups: negative, weak, moderate, and strong. The CREM protein expression is presented as organ system groups below, and the results are summarized in Table 1. The staining intensity evaluation scheme is presented in Fig. 4A, and representative images of the selected tissues are presented in Fig. 4B to P.

**Table 1.** CREM Immunohistochemistry Results in Human Tissues.

Tissue	Area or Cell Type	Dominant Nuclear Staining Intensity	Mean % of Positive Staining Nuclei, (Range)	Comments
Adrenal gland cortex	Cortical cells of zona glomerulosa, fasciculate and reticularis	3	91.5 (87–94)	Moderate CS
Bone marrow	Erythroid cells, cells of granulopoietic lineage, megakaryocytes	0	9.8 (2–23)	
Breast, female, non-lactating	Lobular epithelium	1–2	80.0 (69–91)	
	Ductal epithelium	1–2	76.0 (58–94)	
Bronchus	Respiratory epithelium	1–2	69.0 (68–70)	
Cerebellum^a^	Granular layer	0	1.0 (0–2)	
	Molecular layer	0	0.3 (0–1)	Moderate fibrillar CS
	Purkinje cell layer	0	N/A	
Cerebrum, gray matter^a^	Neurons	0	2.0 (0–5)	
Cerebrum, white matter^a^	Glial cells	0	1.3 (0–3)	
Colon	Enterocytes and goblet cells	1	65.3 (56–71)	
	Stromal cells	2–3	60.5 (58–65)	
Duodenum	Enterocytes and goblet cells	0–1	60.5 (36–77)	
	Stromal cells	1–2	58.0 (54–62)	
	Brunner glands	2	55.5 (53–58)	
Endometrium, proliferative phase	Glands	1	90.0 (90)	
	Stroma	1–2	86.,0 (86)	
Endometrium, secretory phase	Glands	1–2	70.0 (50–90)	
	Stroma	0–1	64.0 (41–87)	
Esophagus	Stratified squamous epithelium	1–2	65.3 (60–70)	
	Stromal cells	3	65.5 (53–73)	
Fimbriae	Columnar ciliated epithelium	3	100.0 (99–100)	
Floor of mouth	Stratified squamous epithelium	1–2	62.0 (53–70)	
Gallbladder	Columnar epithelium	2	79.0 (76–82)	
	Stromal cells	0–2	43.0 (41–46)	
Heart^a^	Myocardiocytes	0	0 (0)	
Kidney	Cells of glomerulus	0–2	51.5 (40–64)	
	Tubular cells	1–2	94.3 (92–97)	
Liver	Hepatocytes	0	0 (0)	Moderate granular CS
	Biliary duct epithelium	0–1	25.0 (0–100)	
Lung	Pneumocytes I and II	2	77.3 (75–80)	
Lymph node cortex	Germinal center	2	85.3 (83–87)	
	Mature lymphocytes	1–2	57.0 (50–62)	
Myometrium	Myocytes	1–2	76.0 (74–78)	
Nasal cavity/Nose	Pseudostratified respiratory epithelium	2	65.8 (60–69)	Goblet cells negative
	Mucinous glands	0	20.0 (19–21)	
	Serous glands	2	82.3 (78–87)	
Ovary	Stroma	0–2	59.0 (48–70)	
	Primary oocytes	2	N/A	Moderate CS
	Granulosa cells	1	N/A	
Palate	Stratified squamous epithelium	2	63.3 (60-–7)	
Pancreas	Islets of Langerhans	1	67.5 (61–72)	Moderate CS
	Exocrine cells	1	74.3 (68–83)	
Parathyroid gland	Endocrine cells	2–3	77.5 (52–88)	
Parotid gland	Ductal epithelium	1–2	73.3 (65–79)	
	Serous glands	1–2	77.8 (71–84)	
Placenta	Cytotrophoblasts	2	51.5 (51–52)	
	Syncytiotrophoblasts	0	0 (0)	
Prostate	Acinar epithelium	1	75.5 (58–93)	
	Fibromuscular stroma	1–2	58.0 (58–60)	
Skeletal muscle	Myocytes	1–2	71.0 (51–82)	Moderate granular CS
Skin	Stratified squamous epithelium	1–2	59.7 (52–65)	
	Melanocytes	0	4.3 (0–11)	
	Eccrine glands	2	86.0 (86)	
	Sebacceous glands	2	99.0 (99)	Moderate CS
Small intestine	Enterocytes and goblet cells	0–1	68.5 (41–89)	
	Stromal cells	1–2	71.0 (62–83)	
Spleen	Red pulp	0–2	54.8 (32–67)	Moderate granular CS
	White pulp	0	34.0 (12–52)	
Stomach	Glandular and superficial epithelium	1–2	63.5 (53–75)	Moderate CS
Submandibular gland	Ductal cells	2	89.5 (83–93)	
	Mucous glands	1	59.3 (58–60)	
	Serous glands	2–3	76.3 (65–85)	
Testis	Seminiferous tubular cells	3	85.0 (74–96)	Strong CS
	Leydig cells	1	N/A	Moderate CS
Thyroid gland	Follicle epithelium	2–3	83.3 (81–85)	Moderate CS
Tongue	Stratified squamous epithelium	0–1	57.0 (21–68)	
	Stromal cells	0–3	46.3 (27–57)	
Tonsil	Stratified squamous cells	1–2	81.3 (74–87)	
	Germinal center	2	62.0 (55–72)	
	Mature lymphocytes	0	32.7 (27–41)	
Urinary bladder	Urothelium	1–2	85.3 (79–91)	
	Stromal cells	2	65.8 (50–83)	

The 39 different tissues are presented in alphabetical order and, when applicable, divided into subgroups by area or cell type. The dominant nuclear staining intensity is reported in a four-tier scale as presented in Fig. 2B. Variation in dominant staining intensity between individual samples is indicated when present. Abbreviations: CS, cytoplasmic staining; N/A, figures not available, under 100 cells of this type per sample.

a Tissue from autopsy.

As a rule, CREM expression was wide, as it was found in almost all tissues. As expected of a transcription factor, CREM was primarily localized in the nuclei of almost all cell types. In most tissues, expression was extensive with positivity in parenchymal cells and supporting structures such as stromal and endothelial cells. Mitotic cells were negative, when present. Mature red blood cells were present in many samples, and they did not express CREM. Most of the tissues had some cytoplasmic staining of different intensities (Table 1).

#### Central Nervous System

In the cerebrum and cerebellum, both cortical neurons and glial cells had virtually no expression of CREM (Fig. 4B). The molecular layer of the cerebellum showed moderate fibrillar staining. Some mild to moderate staining was noted in the endothelial cells of capillaries of the cerebral white matter.

#### Respiratory System

In the nasal cavity, CREM expression in the respiratory epithelium was extensive and of moderate intensity (Fig. 4C). The stroma had moderate to high staining intensity. Below the surface epithelium, both mucinous and serous glands were stained with a moderate intensity. However, the number of cells expressing CREM was dissimilar: in mucinous glands 19–21% and in serous glands 78–87%. The bronchial epithelium had CREM expression of mild to moderate staining intensity. The alveolar epithelium of the lung had extensive CREM expression, in 75–80% of pneumocytes. No significant difference between pneumocytes type I and II was detected.

#### Gastrointestinal Tract and Organs

The stratified non-keratinizing squamous cells of the palate expressed CREM moderately, with positive staining in 60–67% of cells. The nuclei lost their CREM expression as they matured so that usually the upper third or upper quarter of the epithelium was negative (Fig. 4D). The squamous epithelium of the floor of the mouth had a very similar pattern, with slightly weaker expression overall. The same gradient pattern was detected also in the stratified squamous epithelium of the tongue, and the CREM expression varied, with 21–68% of cells being positive with a dominantly low intensity.

Two thirds of the esophageal squamous cell nuclei expressed CREM with a low to moderate intensity. In this epithelium, the positive nuclei were evenly distributed within the epithelium. The stroma had strong expression of CREM, with positivity in 53–73%.

In gastric mucosa, 53–75% of cells expressed CREM. The surface epithelium of the gastric corpus expressed CREM with the exception of apical mucous cells, which were negative. The glandular cells of the gastric corpus showed expression of CREM with weak to moderate intensity (Fig. 4E).

The duodenal epithelium had a mild staining intensity with various range of CREM expression. Enterocytes near the bottom of the crypts tended to be more often negative than apical enterocytes, and Paneth cells were mostly positive. In the stromal cells, we observed a weak to moderate staining intensity in about half of the cell nuclei. Half of the Brunner gland cell nuclei showed CREM expression, and it was of moderate intensity (Fig. 4F). Further along the small intestine, 41–89% of the epithelium expressed CREM with a weak intensity. Goblet cells were negative, and the endocrine cells stained similarly as the enterocytes. In all, 62–83% of the stromal cells had CREM expression of weak to moderate intensity. As in the small intestine, in the colon two thirds of the mucosa had expression of CREM with a weak intensity. Here, the enterocytes in the crypts tended to be more negative than apical cells and the endocrine cells stained similarly as the cryptal enterocytes. Goblet cells were more often negative than the enterocytes. The nuclei in the stroma had moderate to strong staining intensity and a wide expression of CREM.

The acinar and ductal cells in the parotid gland had CREM expression of low to moderate intensity. In the submandibular gland, approximately half of the mucous gland cells had weak staining intensity. On the contrary, nuclei of the serous glands had moderate to strong staining intensity of CREM. Most of the ductal cells showed intensive staining (Fig. 4G).

In the pancreas, both exocrine and the islets of Langerhans endocrine cell nuclei were widely positive with weak intensity (68–83% and 61–72%, respectively). Pancreatic tissue is depicted in fig 4A, as an example of weak staining intensity. In the liver, the hepatocyte nuclei were uniformly negative. Their cytoplasm had some moderate granular staining (Fig. 4H). The biliary duct cells were negative apart from one sample with weak uniform expression of CREM. The gallbladder epithelium consisting of columnar cells had weak to moderate staining in most of the nuclei. Of the stromal cells, less than half of the nuclei had moderate to strong staining intensity.

#### Endocrine Organs

Most of the thyroid follicular cells had moderate to strong staining intensity. In the parathyroid gland, the chief cells and oxyphil cells had a moderate to strong staining intensity in 52–88% of cells. The parathyroid tissue is depicted in Fig. 4A, as an example of strong staining intensity. Adrenal gland cortical cells had a strong and a rather uniform staining pattern.

#### Urinary System

The range of positively staining glomerular cells in the kidney varied from 40% to 60%, and the staining intensity was weak to moderate. The tubular cells were nearly always positive so that the proximal tubules had weak and the distal tubules had moderate staining (Fig. 4I). In the urinary bladder, 79–91% of the urothelial cells had a weak to moderate staining intensity. The stromal cells had a moderate staining CREM staining intensity in 50–83% of nuclei (Fig. 4J).

#### Female Reproductive System

In the non-lactating female breast, the ductal and lobular epithelium stained alike. They had weak to moderate intensity and a near 80% range of CREM expression. The luminal cell nuclei had slightly less intense staining compared with the basal cell nuclei.

In all, 90% of the glands of the proliferative endometrium had weak immunoreactivity. The stroma also showed CREM expression, with weak to moderate staining intensity in 86%. The capillary endothelial cells had a moderate to strong staining intensity and were easily distinguishable in the stroma. A varied expression of CREM was detected in the secretory endometrium. Glandular cells and stroma had weak to moderate staining intensity in 70% and 64% of nuclei, respectively. The smooth muscle cells of the myometrium had a weak to moderate immunoreactivity in 74–78%. The ciliated columnar epithelium of the fallopian tube fimbriae had a strong and uniform staining of the nuclei (Fig. 4K). In the ovaries, the stromal cells stained variably, ranging from negative to moderate intensity. Primordial follicles had uniform moderate CREM expression and the granulosa cells stained partially with moderate intensity. The late third trimester (H38–39) placenta had negative syncytiotrophoblasts. Half of the cytotrophoblasts had moderate-intensity CREM expression in the villi (Fig. 4L).

#### Male Reproductive System

The prostate gland epithelium had weak CREM expression in 58–93% of nuclei. The luminal secretory cells stained with similar intensity. In the seminiferous tubules of the testis, 74–96% of cells were positive with strong intensity. The spermatids had no CREM expression, whereas the Sertoli cells and spermatocytes had staining intensity from negative to strong. Leydig cells had a weak staining intensity (Fig. 4M).

#### Hematopoietic and Lymphatic System

For bone marrow to be sectioned for histological analysis, it is routinely decalcified to make it softer for sectioning. In this way, the bone marrow had an additional step in tissue preparation compared with the other tissues in this study. Bone marrow cell types were identified based on their morphology. Cells of the bone marrow had overall a sparse CREM expression. The erythroid cells were all negative, and the cells of granulopoietic lineage were mainly negative and the megakaryocytes mainly positive with mild staining intensity. Red pulp of the spleen had moderate (to strong) staining intensity of the sinusoidal cell nuclei, whereas the chordal macrophages/pulp chords remained negative. The white pulp had sprinkles of CREM expression of varying intensity (Fig. 4N). The germinal centers of the lymph node cortex had moderate and fairly uniform expression of CREM in contrast to the surrounding cortical mature lymphocytes of which only half had CREM expression of mild to moderate intensity (Fig. 4O). In the tonsil, we observed a similar pattern as in the lymph node; the germinal centers had a much wider and stronger CREM expression than the mature lymphocytes. The non-keratinizing stratified squamous cell epithelium had a positive basal layer with nuclei losing the CREM expression as they matured toward the surface.

#### Skin

The keratinizing stratified squamous cell nuclei in the skin epidermis had mild to moderate immunoreactivity in little over half of the cells. In a vast majority of melanocytes, no CREM expression could be detected. Weak staining was seen in 0–11% of nuclei. Eccrine glands and sebaceous glands showed moderate staining intensity in nearly all of the cell nuclei.

#### Cardiac and Striated Muscle

In myocytes of the heart, we detected no CREM expression. Striated myocytes of the skeletal muscles had extensive expression of CREM with weak to moderate intensity (Fig. 4P).

### CREM Expression in Cancer

**Figure 5.**
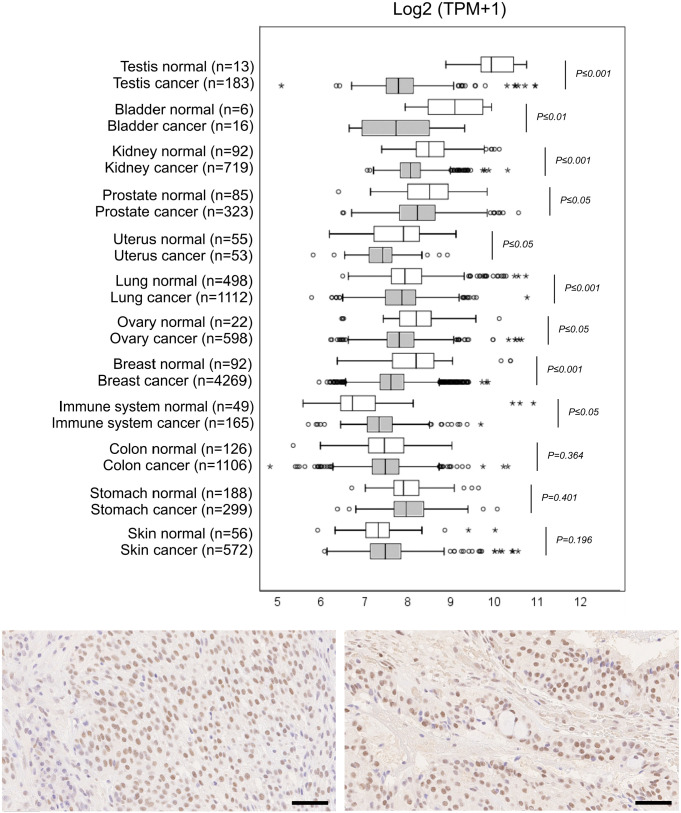
Transcriptomic analysis of CREM in cancer and immunohistochemical staining in mucoepidermoid carcinoma. (A) CREM mRNA expression in human normal tissues and cancer. The logarithmic expression values (log2) of CREM are presented on the *x*-axis as box-plots. The box extends from the first to the third quartile, and the median is presented as a line. The whiskers extend to data points that exist within 1.5× interquartile range (IQR) lower than the first quartile or 1.5× IQR higher than the third quartile. Beyond these values, data points were interpreted as outliers; mild outliers are marked with a circle, whereas extreme outliers are marked with asterisk. Two thirds of the normal tissue versus cancer pairs selected here have downregulation of CREM in cancer tissue; in the rest of the pairs, the corresponding cancer tissues have CREM overexpression. Values of *p* from Student’s *t*-test are indicated. (B) Mucoepidermoid carcinoma with an EWSR1-CREM fusion stained with anti-CREM. Moderate positive staining can be seen homogeneously in a majority of tumor cell nuclei. (C) CREM fusion–negative mucoepidermoid carcinoma stained with anti-CREM. Also here, CREM is positive in most cell nuclei at moderate intensity. Scale bar, 50 µm. Abbreviations: CREM, cyclic AMP element modulator.

Next, we studied whether CREM expression is altered in cancer. For this, we used the GENT2 database (publicly available at http://gent2.appex.kr/gent2/).^18^ In this analysis, we found CREM mRNA expression in all forms of cancer, often with slightly different expression levels compared with normal tissues (Fig. 5A). A slight upregulation was seen in cancers of the immune system and skin cancer. Conversely, in most forms of cancer, downregulation of CREM expression was noted. This was most apparent in cancers of the testis, the urinary bladder, and the breast. Furthermore, there was a large number of outliers in several forms of cancer, with substantially higher or lower expression than in the majority of cases. The difference between CREM mRNA level in normal tissues and cancer was statistically significant in all organs except colon, skin, and stomach.

Second, we wanted to examine whether a tumor with an EWSR1-CREM fusion gene might overexpress the fusion protein, resulting in stronger immunohistochemical CREM staining intensity than seen in the corresponding normal tissues. To test this, five low-grade MECs were immunohistochemically stained and analyzed. One of these had an EWSR-CREM fusion gene, detected by RNA next-generation sequencing and confirmed by RT-PCR using the methodology described previously.^19^ The EWSR1 rearrangement had been confirmed by fluorescence in situ hybridization analysis, using an Abbott Vysis EWSR1 break-apart probe.

In the immunohistochemical analysis, we found moderate positive CREM staining homogeneously in most tumor cell nuclei in all five cases (80–88% of cells). This pattern was slightly different from most normal tissues, in which variation in staining intensity as well as negative nuclei were more often present. The MEC with an EWSR1-CREM fusion gene could not by CREM staining be distinguished from ones that did not harbor this fusion gene (Fig. 5B and C). These findings show that CREM protein is moderately expressed in MEC, and that CREM IHC cannot be used as a surrogate marker in detecting CREM fusion genes in this context.

## Discussion

In this study, we analyzed the expression of CREM protein in 142 samples from 39 tissues. To our knowledge, this is the first systematic study addressing the expression of nearly all CREM isoforms, including the isoform commonly known as ICER. Previously, the Human Protein Atlas (available from http://www.proteinatlas.org) has shown CREM stainings in human tissues using a polyclonal antibody that targets the N-terminal part of the canonical CREM protein expressed almost exclusively in the spermatogenic cells of the testis.^20^ The N-terminal domain of the canonical CREM protein is not present in recently discovered fusion gene products. Given that the antibody used in this study detects almost all CREM isoforms, a wider expression was not an unexpected finding.

We found the expression of CREM in normal human tissues to be wide with only a few exceptions. The staining intensity and differences between cell types within tissues provided more variation and gave more detailed knowledge of CREM expression than mRNA-based tissue analyses. When mRNA levels are studied, a bulk extraction of the tissue is done; thus, it is a mix of all the cell types within the tissue. IHC is much more precise as it can truly show the protein expression of each cell and even the subcellular location. As an example, lymphatic tissues showed moderate expression of mRNA, whereas the protein expression had clear topographic differences. In the lymph node cortex and tonsils, we saw a very clear difference in CREM expression between germinal centers, where expression was strong, and the surrounding mature lymphocytes, which were weak or negative. The expression of CREM in immature B-lymphocytes and loss of expression in mature B-lymphocytes are suggestive of a role in B-cell proliferation and maturation.

At large, the immunohistochemical results were in line with findings from mRNA expression databases. The strongest and most wide staining intensity was seen in reproductive tract tissues: the seminiferous tubules and the fallopian tube fimbrial epithelium. Similar strong staining was also present in salivary gland serous glands, parathyroid glands, and thyroid follicles. Most of the tissues expressed CREM moderately. In the least expressing end of the spectrum, the placental syncytiotrophoblast cells, and the mucus-secreting goblet cells in the epithelial tissues, CREM was virtually undetectable. The mucous glands had less CREM expression profiles than the other cell types of the same tissue. In the liver, hepatocytes had negative nuclei, but a fairly strong granular cytoplasmic staining which could explain the mRNA expression results that indicate positive expression.

Tissues where the immunohistochemical results did not mirror transcription were the CNS and the heart, which had virtually no detectable expression by IHC. This discrepancy may in part be because these tissues were the sole autopsy samples in our study—the inevitable slight delay before fixation of autopsy tissues may have led to degradation of CREM. From other studies we know that CREM is expressed in the CNS, at least regionally. Miller et al conducted an intriguing study in mouse and human where they demonstrated that CREM is a mediator of impulsive action in the basal forebrain, specifically in the nucleus accumbens core (AcbC).^6^ The nucleus accumbens is known for its role in addiction. They further discovered an association between low CREM expression in the AcbC and a higher susceptibility to heroin abuse. Our cerebral samples were, in contrast, all cortical. In another study, the cerebral cortex CREM has been shown to be low, but it increased after neuronal damage.^5^ A wider set of tissue samples would be needed to thoroughly characterize CREM protein expression in the CNS.

In several types of cancer, IHC can be used instead of genetic testing to detect the presence of a specific fusion gene.^21^ EWSR1-CREM gene fusions have recently been found in several types of tumors.^9–13^ We wanted to test whether the baseline expression of CREM in normal tissues would be low enough for the detection of overexpression of a fusion protein. The antibody readily detected an EWSR1-CREM fusion protein in Western blotting of a melanoma cell line, and IHC in an MEC with EWSR1-CREM fusion gene was positive. However, so were MECs without a EWSR1-CREM fusion gene as well as most normal tissues. IHC found positive CREM staining in cell nuclei in virtually all tissues and cell types. With this widely positive background, use of CREM staining for detecting EWSR1-CREM fusion protein in tumors is unlikely a feasible approach.

In transcriptomic analysis, we found that CREM may be clearly upregulated or downregulated in cancer, depending on the tissue type. This divergence is best explained by the dual role of the CREM gene, as it can act as an enhancer or repressor of transcription, depending on which isoform is expressed.^3^ Indeed, isoform-specific expression of CREM has already been linked to prostate cancer; the ICER isoform has been identified as a possible tumor suppressor^22^ and CREM a possible coregulator of apoptosis and carcinogenesis with several differentially expressed miRNAs, affecting prostate cancer progression.^7^ A study in another cancer type, esophageal squamous cell carcinoma (ESCC), found CREM to have prognostic value. Lower CREM expression was associated with a higher risk of metastasis as well as a poorer response to cisplatin, a treatment used in ESCC among many other cancers.^8^ Further studies are warranted to demonstrate the mechanisms behind altered CREM expression, and the consequences thereof, in other forms of cancer.

## Supplemental Material

sj-pdf-1-jhc-10.1369_00221554211032008 – Supplemental material for Expression of Transcription Factor CREM in Human Tissues Click here for additional data file. Supplemental material, sj-pdf-1-jhc-10.1369_00221554211032008 for Expression of Transcription Factor CREM in Human Tissues by Heidi Kaprio, Vanina D. Heuser, Katri Orte, Mikko Tukiainen, Ilmo Leivo and Maria Gardberg in Journal of Histochemistry & Cytochemistry
