# Supplementary material for: Expression of Transcription Factor CREM in Human Tissues
Source: J Histochem Cytochem. 2021 Jul 14;69(8):495–509. doi: 10.1369/00221554211032008 (PMC8329441; doi:10.1369/00221554211032008)
Supplement: sj-pdf-1-jhc-10.1369_00221554211032008 – Supplemental material for Expression of Transcription Factor CREM in Human Tissues [file sj-pdf-1-jhc-10.1369_00221554211032008.pdf]

Supplemental Figure 1

Immunohistochemical detection of CREM in human tissues A) In the cerebellum we detected virtually no nuclear staining. B) In the nasal cavity the serous glands (black arrowhead) were mostly positive and mucous glands (empty arrowhead) mostly negative. Respiratory epithelium (RE).C) The palate squamous epithelium had a gradient expression profile. D) The apical cells (AC) of the gastric corpus were negative while the glandular cells had CREM-expression. E) The duodenum stroma (S) and Brunner glands (B) had weak to moderate staining intensity while the crypts (C) were predominantly negative (unlike the apical enterocytes). F) In the submandibular gland the mucous glands (MG) were weaker and more narrow in their CREM expression the ducts (D) and serous glands (SG). G) In the liver, hepatocytes had negative nuclei, but in the cytoplasm a moderate granular staining was detected. Bile ductile (Bi) with no CREM expression. H) In the kidney the glomerulus (G) only just over half of the nuclei stained positive, the proximal tubules (PT) and distal tubules (DT) had wider expression. I) In the urinary bladder epithelium most of the nuclei had CREM expression. J) The ciliated columnar cells of the fimbriae epithelium were among the strongest staining cell types. K) Here the secondary and tertiary villi of a late third trimester placenta have negative syncytiotrophoblasts lining the villi while half of the cytotrophoblasts in the middle have moderate CREM expression. L) In the testis seminiferous tubules (T) had varying nucleal staining intensity from negative to strong while Leydig cells (L) were mostly positive with mild to moderate intensity. M) The white pulp (W) in the spleen was mostly negative, while in the red pulp (R) we observed clear staining of the sinusoidal cell nuclei. N) In the lymph node cortex the germinal center (G) had a near uniform positive CREM expression while interestingly the mature cortical lymphocytes (M) remained rather negative. O) Skeletal muscle nuclei had a wide weak to moderate staining pattern. Here the capillaries and supporting tissue is rather negative. The scale bar in picture O is 50µm, all images are of the same magnification.

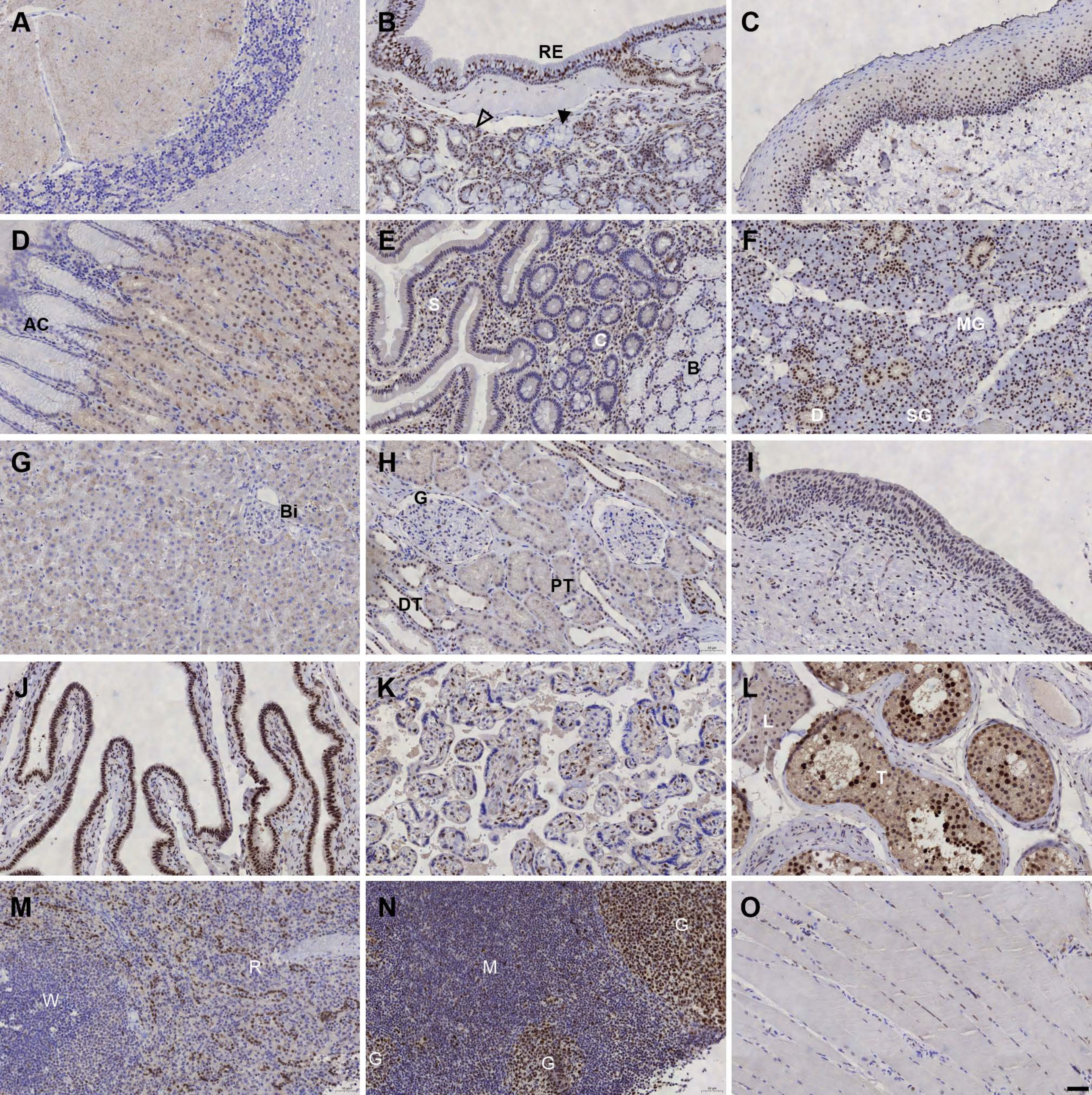
A
B
RE
C
D
AC
E
S
C
B
F
MG
D
SG
G
Bi
H
G
PT
DT
I
J
K
L
L
T
M
R
W
N
M
G
G
G
O
